# Variability and interpretability of benzylpenicillin inhibition zone edges in *Staphylococcus aureus* – a multi-center study in Austria

**DOI:** 10.1007/s10096-025-05374-4

**Published:** 2025-12-23

**Authors:** Felix Lötsch, David N. Springer, Brigitte Selitsch, Sonja Lener, Barbara Ströbele, Lamiss Mejdoubi, Philipp Grubwieser, Silke Huber, Dieter Mitteregger, Harald Dirschmid, Verena Schliesser, Alexandra Wojna, Markus Hell, Birgit Willinger

**Affiliations:** 1 Division of Clinical Microbiology, Clinical Institute of Laboratory Medicine, Medical University of Vienna, Währinger Gürtel 18-20, Vienna, 1090 Austria; 2 Comprehensive Center for Infection Medicine, Medical University of Vienna, Währinger Gürtel 18-20, Vienna, 1090 Austria; 3 Center for Virology, Medical University of Vienna, Kinderspitalgasse 15, Vienna, 1090 Austria; 4 Department of Microbiology, Mühl-Speiser-Bauer-Spitzauer and Partner Labors.at OG, Kürschnergasse 6B, Vienna, 1210 Austria; 5 Clinical Institute for Hygiene and Microbiology, University Hospital St. Pölten, Dunantplatz 1, St. Pölten, 3100 Austria; 6 Department of Clinical Microbiology, Institute of Pathology and Microbiology, Hanusch Hospital, Heinrich-Collin Strasse 30, Vienna, 1140 Austria; 7 Institute of Hygiene and Medical Microbiology, Medical University of Innsbruck, Schöpfstrasse 41, 6020 Innsbruck, Austria; 8 Department of Clinical Microbiology, Institute of Clinical Pathology, Cytology and Microbiology, Dr. Kosak GmbH, Mariannengasse 14, Vienna, 1090 Austria; 9 Institute for Pathology, Landeskrankenhaus Feldkirch, Carinagasse 47, Feldkrich, 6800 Austria; 10 Institute of Laboratory Diagnostic and Microbiology, Klinikum Klagenfurt am Wörthersee, Feschnigstrasse 11, Klagenfurt, 9020 Austria; 11 Department of Clinical Microbiology and Hygiene, MEDILAB-Labor- GesmbH, Academic Teaching Laboratory of Paracelsus Medical University (PMU)-Salzburg, Strubergasse 20, Salzburg, 5020 Austria

**Keywords:** Staphylococcus aureus, Benzylpenicillin, EUCAST, Antimicrobial susceptibility testing, Austria

## Abstract

**Purpose:**

Benzylpenicillin is regaining attention as a treatment option for susceptible *S. aureus*, including in severe invasive diseases such as blood stream infections. Timely and reliable susceptibility determination is essential to support its use in clinical practice. In this study, we assessed the EUCAST-recommended methodology of interpreting zone edges in a national multicenter trial.

**Methods:**

In total, nine microbiology laboratories in Austria participated. Each center received 10 isolates in blinded duplicates, all with inhibition zones of ≥ 26 mm. Three were *blaZ*-positive with sharp edges and seven were *blaZ*-negative with fuzzy edges. Benzylpenicillin susceptibility testing according to EUCAST guidelines using 1 unit discs was performed by two independent technicians in duplicate on two separate days. All plates were interpreted by two different assessors generating a total of 1440 data points.

**Results:**

Overall, 85.5% of all interpretations were correct. Both, major and very major errors occurred. There was high variability between laboratories with overall accuracy ranging from 61.9% to 100%. There were statistically significant differences in the average inhibition zone between MH agars from different manufacturers. Laboratories using agars giving rise to a smaller average inhibition zone (i.e. lower diffusion of benzylpenicillin) performed less well. Laboratories with the methodology implemented performed better than those without experience.

**Conclusion:**

In summary, our study demonstrates that benzylpenicillin susceptibility testing using the EUCAST-recommended methodology is feasible, but accurate and reproducible assessment of zone edges requires both experience and optimal materials. Thorough validation of locally used materials is therefore essential before implementing this approach for routine use.

**Supplementary Information:**

The online version contains supplementary material available at 10.1007/s10096-025-05374-4.

## Introduction

*Staphylococcus aureus* is one of the most frequent bacterial pathogens and may cause life-threatening infections including bloodstream infections and endocarditis. Antimicrobial treatment decisions traditionally depend on the differentiation between methicillin-susceptible (MSSA) and methicillin-resistant (MRSA) *S. aureus*, mediated by the production of an alternative penicillin-binding protein PBP2a encoded in *mecA* or *mecC* genes [1]. Most microbiology laboratories are well equipped to rapidly discriminate between MSSA and MRSA by phenotypic and/or molecular methods. For the treatment of MSSA, guidelines recommend either penicillinase-stable penicillins, such as flucloxacillin or oxacillin, or first-generation cephalosporins, such as cefazolin [2]. However, *S. aureus* is not intrinsically resistant to benzylpenicillin. Recently, a rise in benzylpenicillin-susceptible isolates has been reported from various groups around the globe [3]. Additionally, preliminary data from a multinational, randomized controlled trial (SNAP trial) presented at ESCMID 2025 (abstract L0033 | 07777) show that benzylpenicillin is at least equal – and probably even superior – in treating *S. aureus* bloodstream infections compared to flucloxacillin or oxacillin, when tested susceptible [4]. Retrospective, observational data also support the role of benzylpenicillin in infections caused by *S. aureus* [5]. This highlights the requirement for microbiology laboratories to reliably determine benzylpenicillin-susceptibility in clinical MSSA isolates.

Benzylpenicillin-resistance in *S. aureus* is usually and most importantly conferred by production of a penicillinase [6], which is coded in the *blaZ* gene. However, determination of the mean inhibitory concentration or the inhibition zone diameter alone are not sufficient for the detection of penicillinase producers [7]. EUCAST therefore recommends disk diffusion in combination with inspection of the zone edge. Isolates with a sharp zone edge (like a cliff; “punched out”) are penicillinase producers and need to be reported as resistant despite a large inhibition zone of ≥ 26 mm. Isolates with an inhibition zone of ≥ 26 mm and a fuzzy zone edge (like a beach) are reported susceptible [8]. Due to the subjective nature of the qualitative interpretation of the zone edge, concerns about suitability for routine laboratories were raised. A multi-center trial was initiated in Austria to investigate the applicability of the method in clinical microbiology laboratories, the reasons and factors influencing the reliability as well as the reproducibility of this method before implementation at our institution.

## Materials and methods

We invited 16 Austrian microbiology laboratories to this reproducibility trial. Nine laboratories from five provinces ultimately participated. The Division of Clinical Microbiology of the Medical University of Vienna was the coordinating institution. We planned to distribute to each laboratory nine subsequent clinical isolates with a benzylpenicillin disk diffusion zone of ≥ 26 mm and the ATCC12600 strain, which is benzylpenicillin-susceptible. However, as eight out of nine subsequent isolates were *blaZ*-negative (and therefore benzylpenicillin-susceptible) we replaced two *blaZ*-negative isolates by two *blaZ*-positive strains with an inhibition zone of ≥ 26 mm from our strain collection (see Table 1). All isolates were tested by using the EUCAST-recommended phenotypic method [8] and molecular methods at the Division of Clinical Microbiology of the Medical University of Vienna. For molecular testing, we used a previously published PCR protocol [9]. In all isolates, the phenotypic appearance of the zone edge corresponded to the molecular results (*blaZ*-positive and sharp edge; *blaZ*-negative and fuzzy edge) according to experts at the coordinating reference center.

**Table 1:** Isolate used in this multi-center trial including the *blaZ*-status and phenotypic zone edge appearance

Isolate	blaZ status	zone edge
1 (ATCC12600)	negative	fuzzy
2	negative	fuzzy
3	positive	sharp
4	positive	sharp
5	positive	sharp
6	negative	fuzzy
7	negative	fuzzy
8	negative	fuzzy
9	negative	fuzzy
10	negative	fuzzy

All strains had been stored at −80 °C and were for study purposes inoculated individually on Columbia sheep blood (CSB) agar plates (BD Columbia III 5% SB). After incubation over night at 36 °C in ambient air, the strains were transferred to new CSB agar plates and incubated again. On the following day, individual colonies were suspended in brain heart infusion broth (ThermoScientific brain heart infusion Broth) and split into aliquots to be distributed to each center. Each strain was sent in duplicate to the participating centers. Participants were blinded to the *BlaZ* status and duplicates. In summary, each center received 20 suspensions from 10 unique strains. Participants were instructed to inoculate each one individually on a CSB agar plate, to incubate the plates over night at 36 °C, and to perform benzylpenicillin disk diffusion susceptibility testing according to EUCAST recommendations. All centers were asked to use their locally implemented materials (Table 2) and received no specific training regarding the interpretation of the zone edge, but were referred to materials provided by EUCAST [8].

**Table 2:** Materials used by each participating center

Center	MH agar	Disk
Center 01	MH agar 1	Disk 1
Center 02	MH agar 1	Disk 1
Center 03	MH agar 2	Disk 2
Center 04	MH agar 1	Disk 1
Center 05	MH agar 1	Disk 2
Center 06	MH agar 1	Disk 2
Center 07	MH agar 2	Disk 3
Center 08	MH agar 4	Disk 1
Center 09	MH agar 3	Disk 4

*MH agar* *1* BioMérieux Mueller-Hinton-E-Agar; *MH agar* *2* BD BBL Mueller-Hinton II agar; *MH agar* *3* BD BBL Mueller-Hinton-II-Agar - in-house made; *MH agar 4* Thermo Fisher Scientific Mueller Hinton agar; *Disk 1* OXOID Penicillin G 1 unit; *Disk 2* MASTDISCS® Penicillin G 1 unit; *Disk 3* SIRSCan® Penicillin G 1 unit; *Disk 4* BD BBL™ Sensi-Disc™ Penicillin G 1 unit

Antimicrobial susceptibility testing of each aliquot was conducted by two independent technicians in duplicate and the procedure was repeated on a second day. Plate reading and interpretation of the zone was performed by two independent readers. In summary, 16 data points were thus generated per isolate per center (leading to 1440 data points in total).

To better understand the factors underlying the fuzzy edge phenomenon, we created a simplified simulation of the diffusion of benzylpenicillin from the disk into the agar and its neutralization by the *blaZ*-encoded penicillinase. In this simulation, both benzylpenicillin and penicillinase were modeled to decrease exponentially with distance from their source with both decay rates modified by an agar diffusion resistance factor (for details, see Supplementary Methods). Penicillinase production was assumed to begin at the point where benzylpenicillin concentration first fell below the minimal inhibitory concentration (MIC). The inhibitory effect of benzylpenicillin activity was represented by a simple multiplicative term, reducing effective benzylpenicillin concentration in proportion to local penicillinase concentration. While this simplified model ignores time dynamics and complex reaction-diffusion interactions, it allows for an intuitive exploration of how decay rates, agar resistance, inhibition strength and MIC thresholds influence the inhibition zone.

Statistical analyses were performed using R with the following packages: *dplyr*, *tidyr*, *ggplot2*, *lubridate*, *viridis*, *RColorBrewer*, *ggsci*, *forcats*, *cowplot*, *rpart*, *rpart.plot*,* partykit* and *shiny*. During the preparation of this work the authors used the LLM Chat GPT 4.0, OpenAI in order to solely enhance grammar and sentence clarity, without contributing to the creation or modification of the scientific content in this manuscript. After using this tool, the authors reviewed and edited the content as needed and take full responsibility for the content of the publication. The study was performed between 19 May 2025 and 27 June 2025.

## Results

**Fig. 1:**
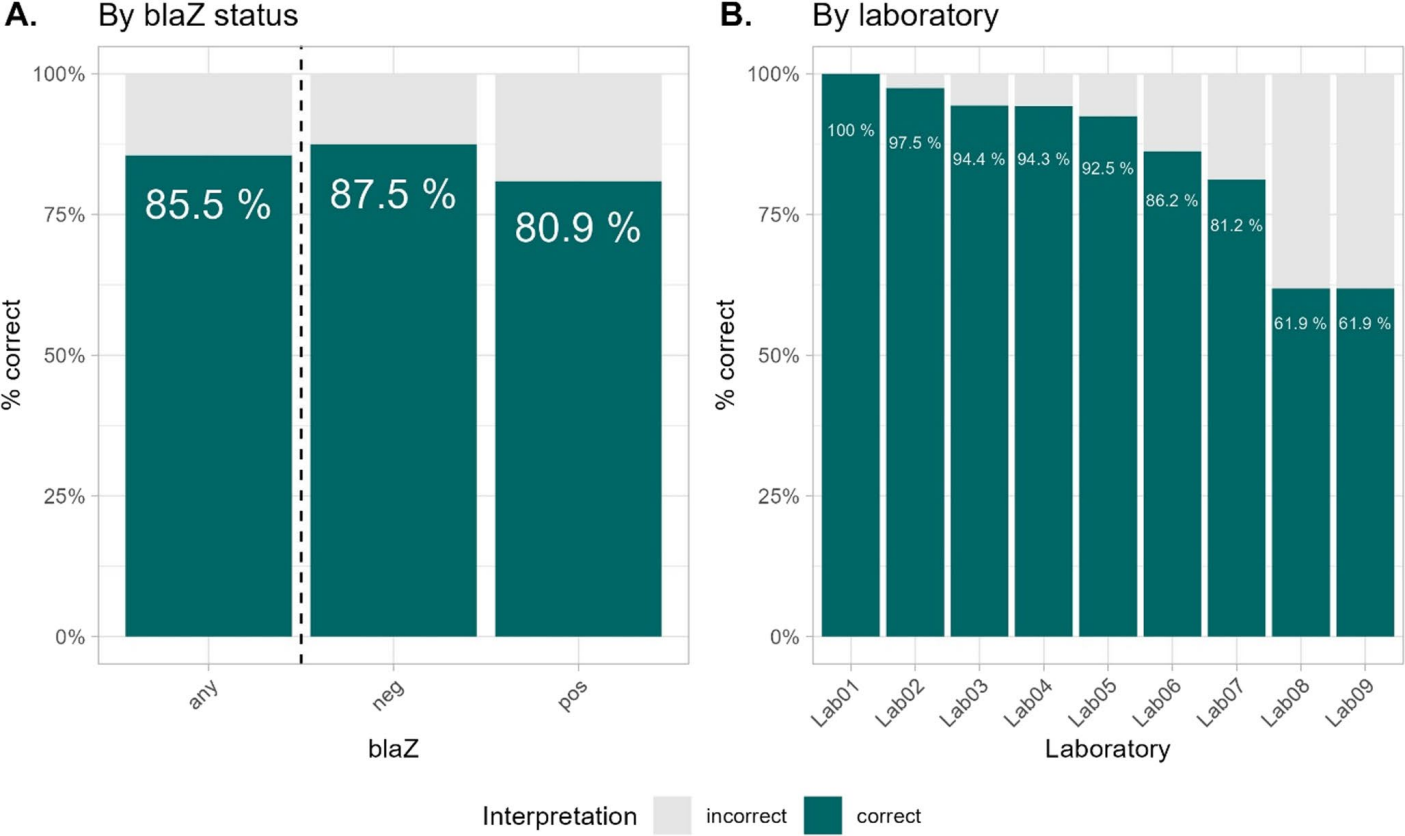
Panel A shows the proportion of correct interpretations of the inhibition zone edge („any“) according to blaZ status. Panel B shows the proportion of correct reads per laboratory

1438 data points from 10 strains were created by 9 different centers. In one center, one strain did not show growth on a single day, leading to two missing data points. Overall, 85.5% of all reads were correct with a range from 61.9% to 100% per center (see Fig. 1). There were significantly more errors in *blaZ*-positive isolates (80.9% correct reads) than in *blaZ*-negative isolates (87.5% correct reads) (*p* = 0.002). The proportion of correct reads was significantly higher in laboratories with the disk diffusion method of benzylpenicilline susceptibility testing of *S. aureus* implemented (89.6% correct reads) compared to those which are currently not using the method in their routine work (80.5% correct reads; *p* < 0.001).

**Fig. 2:**
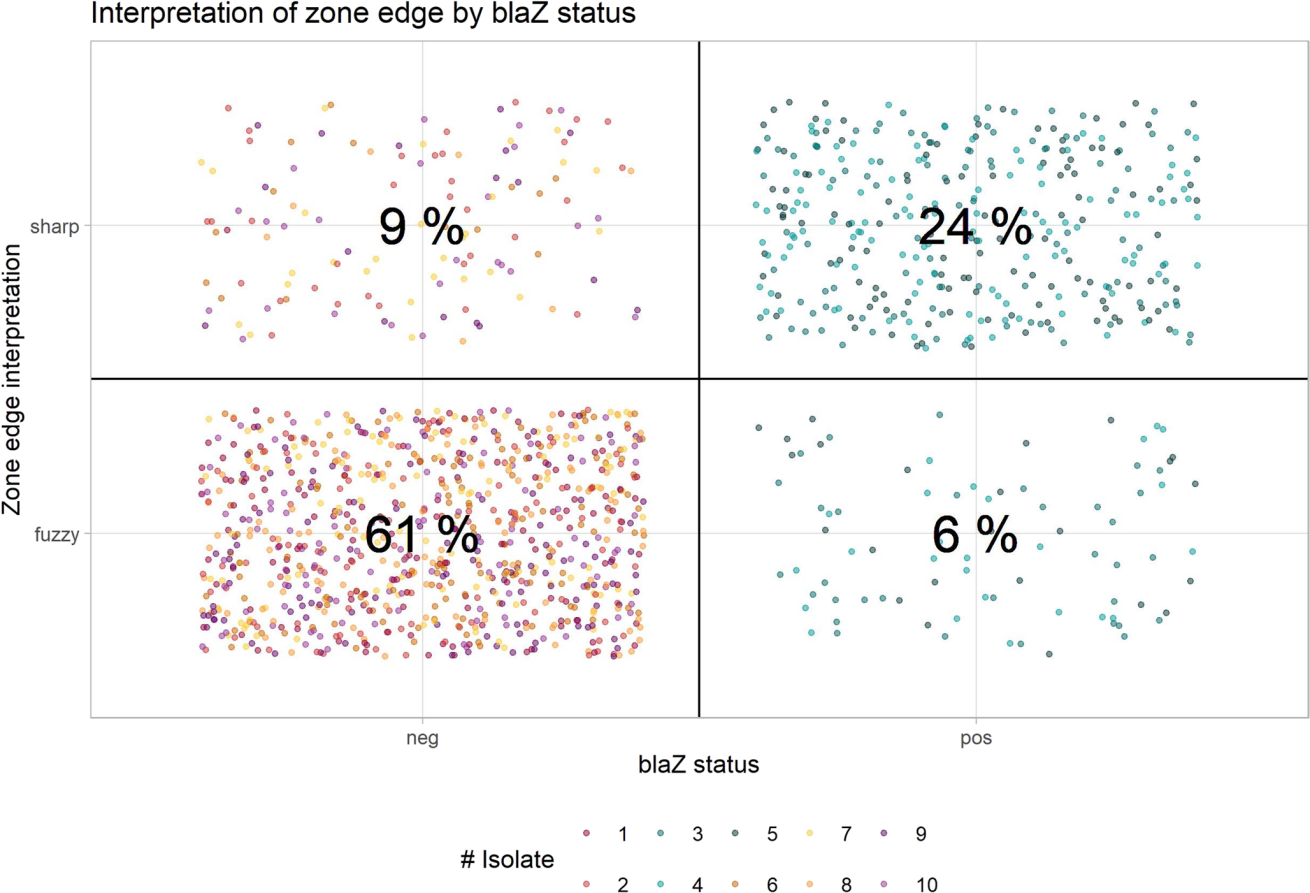
Plot showing correct interpretations of inhibition zone edges, major errors (9%) and very major errors (6%). Percent refer to the overall number of intrepretations (*n* = 1438)

Both major (“fuzzy”/susceptible interpreted as “sharp”/resistant) and very major (“sharp”/resistant interpreted as “fuzzy”/susceptible) errors occurred (see Fig. 2).

**Fig. 3:**
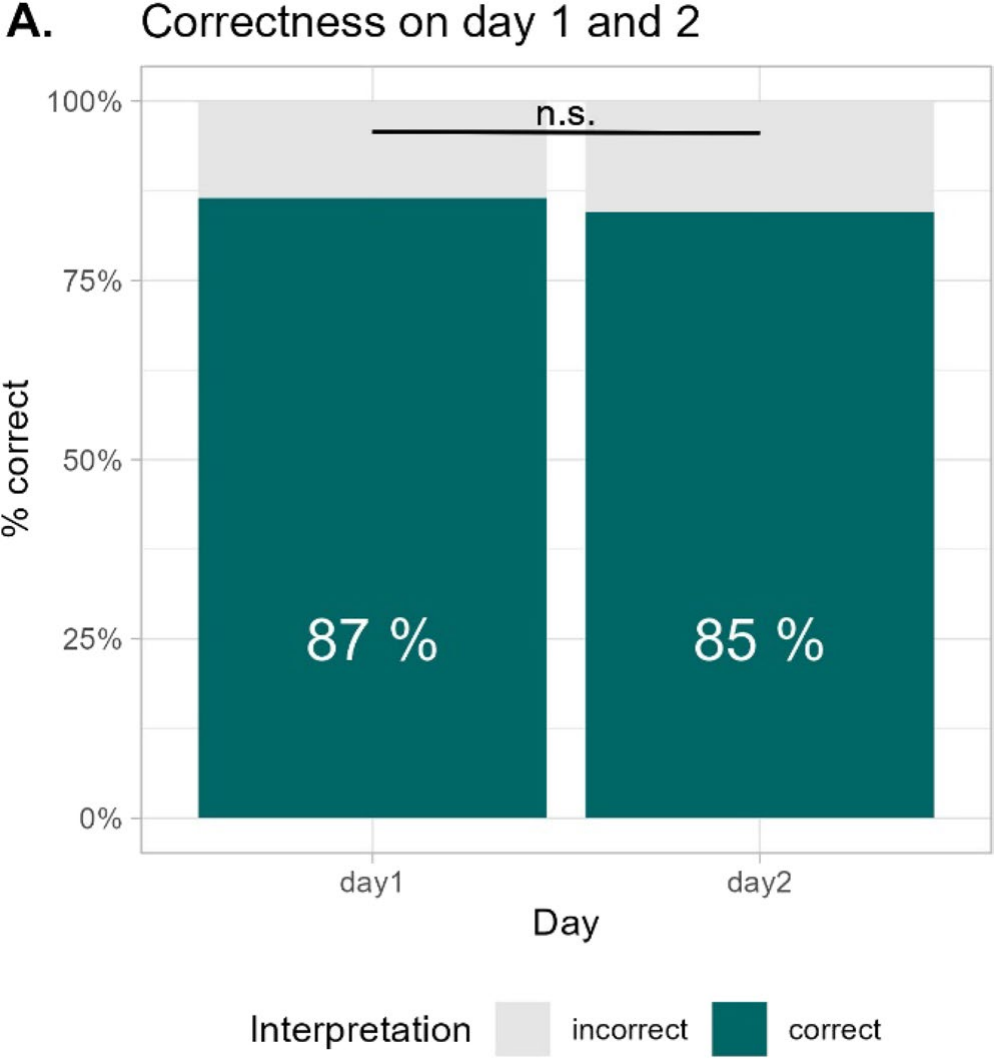
Panel A shows the proportion of correct interpretations per day across all laboratories

There was variability in the proportion of correct interpretations between isolates. While isolate 1 (ATCC12600) was interpreted correctly in 99% of reads, only 76% were correct in isolate 2 (see supplementary Fig. 1). 4 out of 9 centers had no “very major error” (*blaZ*-positive isolates interpreted as “fuzzy”, i.e. susceptible) while only one center had neither a “major error” (*blaZ*-negative isolates interpreted as “sharp”, i.e. resistant) nor a “very major error”. Agreement between readers over all centers was substantial (Cohen’s Kappa 0.71) with a wide variability between participating centers ranging from a κ of 0.34 (fair agreement) to 1.00 (perfect agreement). Comparing the correctness between day 1 (87%) and day 2 (85%) to assess a possible training effect showed no difference (*p* = 0.99) (see Fig. 3). Only one laboratory performed better on day 2 (laboratory 06).

**Fig. 4:**
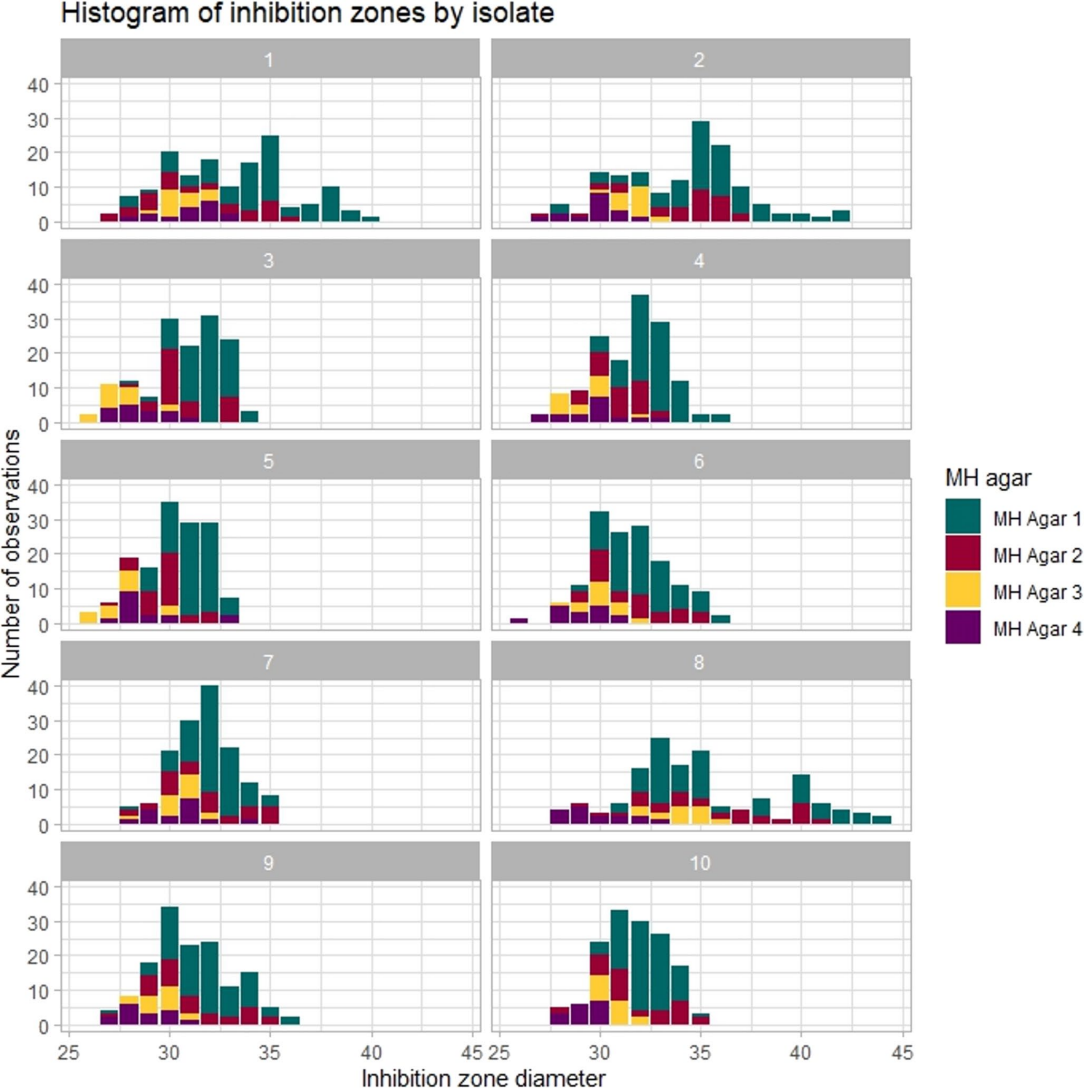
Histogram of disk diffusion zones per MH agar. MH agar 1 = BioMérieux Mueller-Hinton-E-Agar; MH agar 2 = BD BBL Mueller-Hinton II agar; MH agar 3 = BD BBL Mueller-Hinton-II-Agar - in-house made; MH agar 4 = Thermo Fisher Scientific Mueller Hinton agar. MH agar 1 and 2 tend to result in larger inhibition zones than MH agar 3 and 4

Median inhibition zones differed significantly depending on the agar used. The largest zones were produced by MH Agar 1 (BioMérieux Mueller-Hinton-E-Agar) (median 32 mm, 25th to 75th percentile 31–34 mm) and MH Agar 2 (BD BBL Mueller-Hinton II agar) (median 31 mm, 25th to 75th percentile 30–34 mm). Median disk diffusion zones of MH Agar 3 (BD BBL Mueller-Hinton-II-Agar - in-house made) was 30 mm (25th to 75th percentile 29–31 mm) and of MH Agar 4 (Thermo Fisher Scientific Mueller Hinton agar) 29.5 mm (25th to 75th percentile 28–30 mm) (see supplementary Fig. 6). A histogram of disk diffusion zones per isolate and MH agar is shown in Fig. 4. The overall proportion of correct reads was 94% for MH Agar 1 (BioMérieux Mueller-Hinton-E-Agar), 88% for MH Agar 2 (BD BBL Mueller-Hinton II agar), 62% for MH Agar 3 (BD BBL Mueller-Hinton-II-Agar - in-house made) and 62% for MH Agar 4 (Thermo Fisher Scientific Mueller Hinton agar). Correct reads per disk used was 91% for benzylpenicillin disk 1 (OXOID Penicillin G 1 Unit), 88% for benzylpenicillin disk 2 (MASTDISCS Penicillin G 1 Unit), 81% for benzylpenicillin disk 3 (SIRSCan^®^ Penicillin G 1 unit) and 62% for benzylpenicillin disk 4 (BD BBL™ Sensi-Disc™ Penicillin G 1 unit) (see supplementary Fig. 3).

**Fig. 5:**
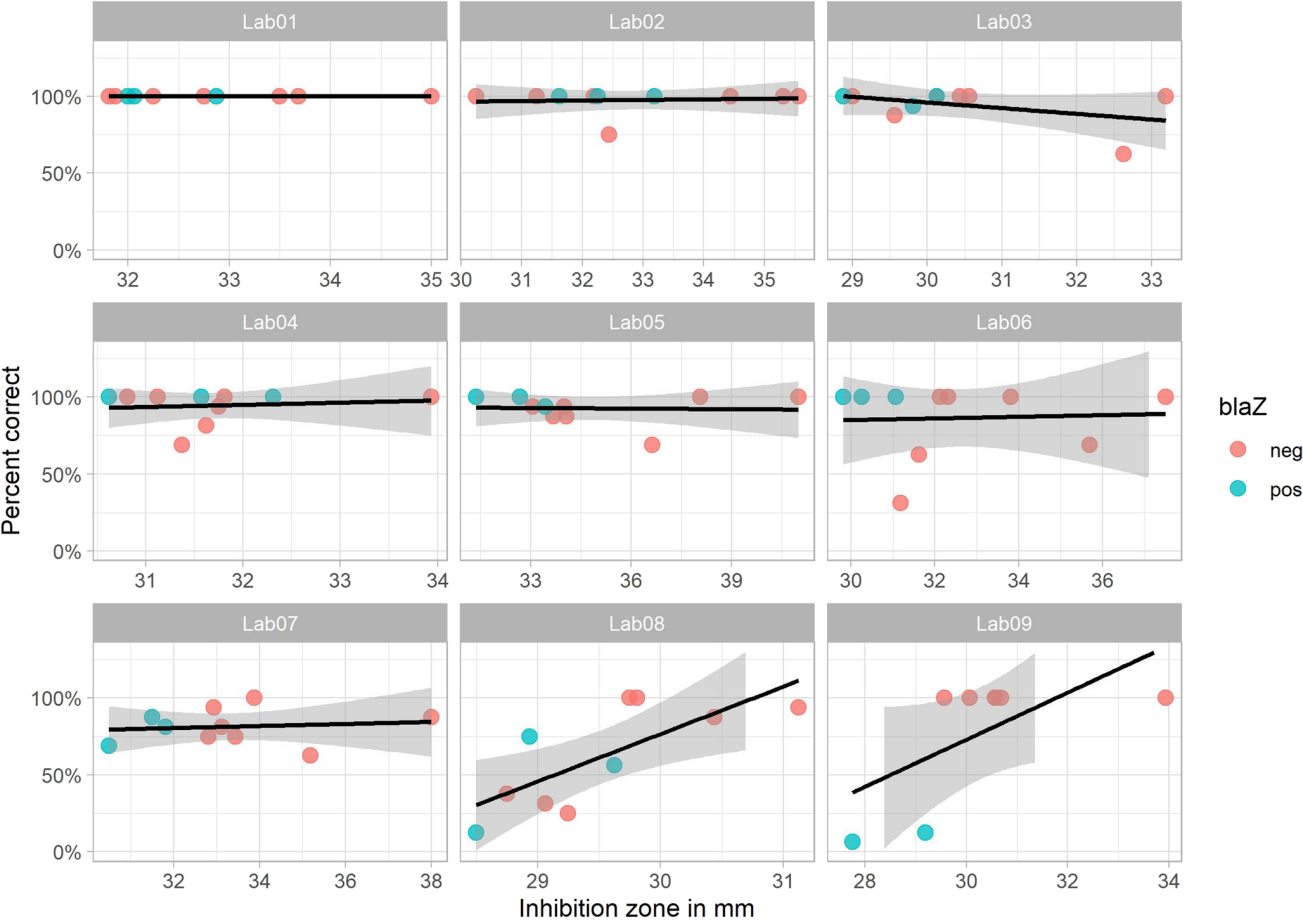
Proportion (%) of correct reads with mean inhibition zone per center. Each isolate (1 to 10) is shown separately

Correct interpretation of the zone edge in individual isolates appeared to correlate with the inhibition zone (see Fig. 5). To dissect which variables performed best at classifying into correct or incorrect reads, a decision tree was built including the variables MH agar, benzylpenicillin disk, *blaZ*-status and routine implementation of using disk diffusion for susceptibility testing of *S. aureus*. With a complexity parameter set to 0.01, only MH agar and *blaZ*-status were useful for classification (see supplementary Fig. 7). Using MH agar 1 (BioMérieux Mueller-Hinton-E-Agar) or 2 (BD BBL Mueller-Hinton II agar) versus using MH agar 3 or 4 was the best variable for splitting correct from incorrect reads. When using agar 3 (BD BBL Mueller-Hinton-II-Agar - in-house made) or 4 (Thermo Fisher Scientific Mueller Hinton agar), *blaZ* positive isolates were more often incorrectly interpreted than *blaZ* negative isolates.

Ultimately, we simulated the transition zone (“fuzzy” vs. “sharp”) in a mathematical model with different values for the assumed agar diffusion capacity. Increasing the diffusion capacity expanded the transition zone in *blaZ*-negative isolates improving the readability and interpretability (see supplementary Figs. 8 and 9).

**Fig. 6:**
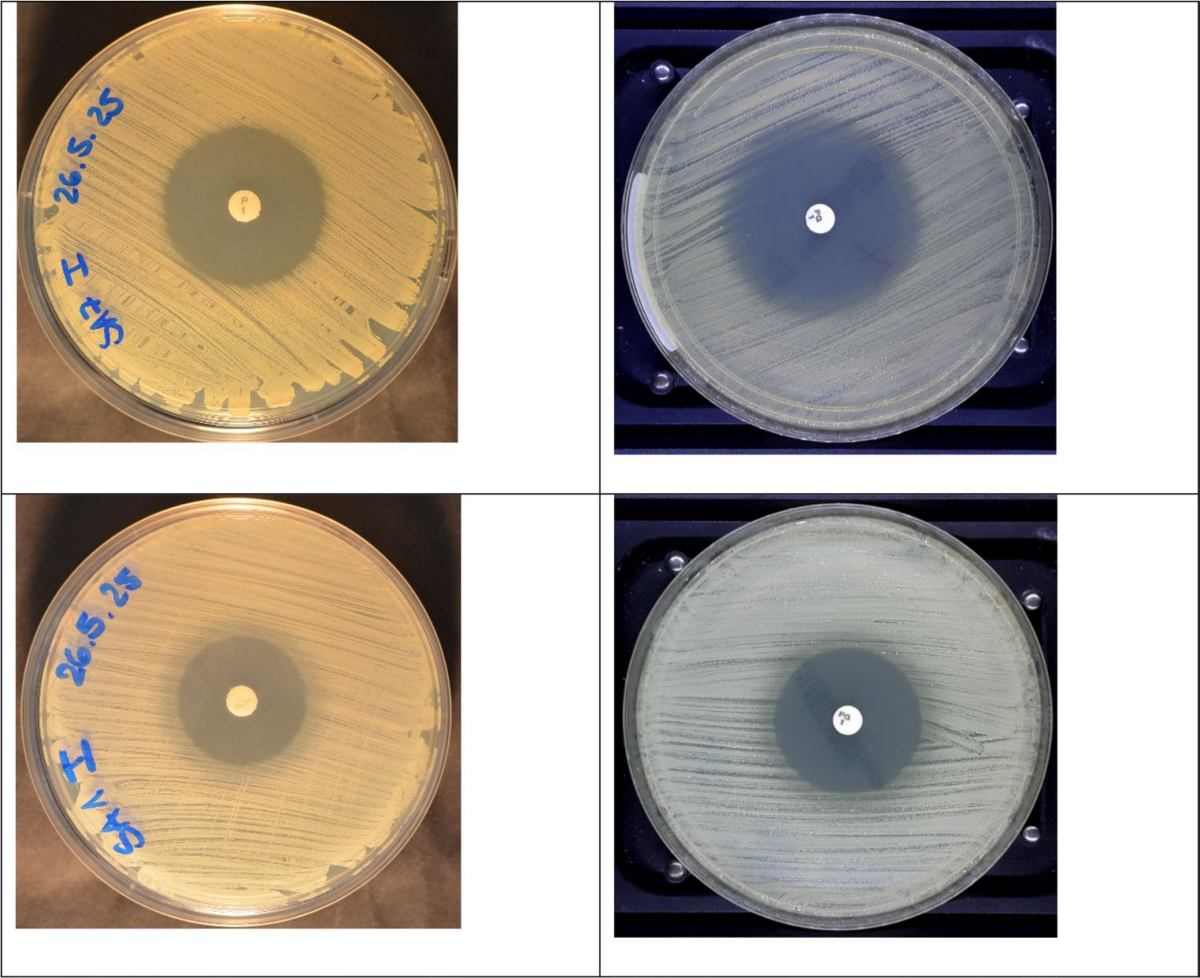
The upper images show the identical benzylpenicillin-susceptible isolate (fuzzy edge), the lower images the identical benzylpenicillin-resistant isolate (sharp edge). Images on the left use MH agar 3 (BD BBL™ Mueller-Hinton-II-Agar; in-house made), images on the right MH agar 1 (BioMérieux Mueller-Hinton-E agar)

Furthermore, to test the conclusions drawn, pictures taken from benzylpenicillin susceptibility testing from each aliquot of the 10 strain suspensions using MH agar 1 (BioMérieux Mueller-Hinton-E-Agar) and benzylpenicillin disk 1 (OXOID Penicillin G 1 Unit) (see right panel of image 1) were sent to each center and interpreted by two independent assessors. 354 out 360 interpretations (98.3%) were correct. Especially in *blaZ*-positive isolates, it was observed that a rim of thinner growth appeared before the complete (innermost) end of growth followed towards the center of the inhibition zone. It was important not to interpret this thinner growth as “beach”, since only the innermost end of growth should be investigated to avoid major errors (see also lower panel of Fig. 6).

## Discussion

Benzylpenicillin is regaining attention as a treatment option for benzylpenicillin-susceptible *S. aureus*, including severe invasive disease such as blood stream infections. Timely and reliable susceptibility determination is essential to support its use in clinical practice. According to EUCAST guidelines [8], interpretation should include both measurement of the inhibition zone diameter by disk diffusion and assessment of the zone edge. While determination of the inhibition zone diameter is generally straightforward, evaluation of the zone edge seems more challenging. In this study, we demonstrated that reproducible and accurate interpretation of the zone edge in benzylpenicillin susceptibility testing of *S. aureus* is achievable, but is contingent on optimal testing conditions. Five out of nine laboratories correctly interpreted more than 90% of readings, with one laboratory achieving 100% accuracy. In contrast, two experienced institutions, both with specialist microbiology staff and established disk diffusion methodology, achieved only 61.9% correct interpretations. Notably, these two laboratories used MH agar from manufacturers different from those used by all other participating laboratories (BD BBL Mueller-Hinton-II-Agar - in-house made and Thermo Fisher Scientific Mueller Hinton agar). MH agars from BioMérieux (BioMérieux Mueller-Hinton-E-Agar) and ready-to-use agar from BD (BD BBL Mueller-Hinton II agar) were associated with correct reads.

Interestingly, the average inhibition zones observed on these agars were significantly smaller than those obtained with other MH agars used by higher-performing laboratories. This may be attributable to reduced diffusion of benzylpenicillin - the active growth-inhibiting agent - through the agar, resulting in smaller zones of inhibition. Mathematical modeling suggests that reduced diffusion compresses the transition zone of faint growth in *blaZ*-negative isolates (“fuzzy edge”), thereby making their phenotypic appearance more closely resemble that of *blaZ*-positive isolates (“sharp edge”). This convergence complicates the visual distinction between “sharp” and “fuzzy” edges (see Fig. 6). The association is further supported by analysis of individual isolates, which shows a correlation between smaller average inhibition zones and reduced accuracy of interpretation.

The correctness of readings appeared to correlate negatively with the average disk diffusion zone diameter (Fig. 5). While one of the two lower-performing laboratories used a unique benzylpenicillin disk, the other employed the same disk as the best-performing laboratory, suggesting that the antibiotic disk itself is unlikely to account for the observed differences. In a regression tree analysis, the model selected the type of MH agar, rather than the disk, as the primary variable distinguishing correct from incorrect interpretations. Nonetheless, a potential interaction between disk and agar cannot be excluded, but this was beyond the scope of the present study.

Both major and very major errors were observed, which is unsurprising given that, depending on the materials used and the strain tested, “sharp” and “fuzzy” zone edges can appear very similar (e.g. on MH agar three as shown in Fig. 6). Consequently, resistant isolates (expected to display a sharp edge) may be misclassified as fuzzy, and vice versa. Laboratories that had already implemented the zone-edge assessment methodology - either for all *S. aureus* isolates or upon specific request - generally achieved higher accuracy than laboratories without such experience. However, no difference in performance was observed between days 1 and 2, indicating that accurate and reliable application of the method requires prior experience and training, and that brief exposure (e.g., one day) is insufficient to achieve a training effect. At last, interpretation of benzylpenicillin zone edges from images of each aliquot using the best performing agar and benzylpenicillin disk achieved a correctness of 98.3% demonstrating excellent interpretability. This highlights the importance of using optimal materials.

This study has several limitations. Its primary aim was to evaluate whether the zone-edge interpretation methodology is suitable for routine implementation in clinical microbiology laboratories, and it was not designed to compare different disks or agars in a head-to-head fashion across a sufficiently large number of isolates. Both laboratories that underperformed used unique agars, and we lack data on how these agars would perform in other laboratory settings. Our findings should therefore be interpreted with caution and confirmed in a dedicated investigation. Additionally, we did not include the ATCC29213 reference strain recommended by EUCAST for quality control of the disk diffusion methodology. The reason was that this is a benzylpenicillin-resistant isolate with a small inhibition zone. Such isolates usually do not pose problems as they are easily recognized as resistant. They were therefore not subject of this study. In retrospective, it would have been interesting to confirm the association between agar and inhibition zone diameter found in our study in this reference quality control isolate.

However, to the best of our knowledge, this is the first study to identify and compare performance differences in benzylpenicillin susceptibility testing of *S. aureus* attributable to the materials used, particularly the type of agar. Previous investigations were predominantly single-center studies, typically conducted in experienced reference laboratories, and focused on comparing methods for benzylpenicillin susceptibility testing [7, 10, 11]. Another strength is that all laboratories were blinded to the *blaZ* status of the isolates and not influenced by results from alternative methods (e.g. PCR). Unfortunately, no official reference strains are available for quality control (QC) purposes in terms of this phenotypic method. Laboratories therefore have to create their own QC isolates using molecular tests as reference methods, but these are also insufficiently standardized. Reference strains defined by respective authorities (e.g. EUCAST) are therefore urgently required.

In summary, our study demonstrates that benzylpenicillin susceptibility testing using the EUCAST-recommended methodology is feasible, but its accuracy and reproducibility depend on both user experience and the use of suitable materials. Performance varied substantially between laboratories, with the best results achieved in those that had prior experience with zone-edge interpretation and used specific MH agars (bioMérieux Mueller-Hinton-E-Agar or ready-to-use BD BBL Mueller-Hinton II agar) that supported larger inhibition zones. In contrast, laboratories using alternative agar formulations obtained smaller zones and more ambiguous edges, which can increase the risk of misclassification. These findings underline the need for careful and extensive validation of all locally used materials - particularly agar type - before adopting the methodology for routine diagnostics, as well as targeted training to ensure consistent interpretation across users.

## Supplementary Information

Below is the link to the electronic supplementary material.

Supplementary file1 (DOCX 912 KB)

## Data Availability

The datasets generated during and/or analyzed during the current study are available from the corresponding author on reasonable request.
