# Supplementary material for: Variability and interpretability of benzylpenicillin inhibition zone edges in Staphylococcus aureus – a multi-center study in Austria
Source: Eur J Clin Microbiol Infect Dis. 2025 Dec 23;45(4):977–86. doi: 10.1007/s10096-025-05374-4 (PMC13086644; doi:10.1007/s10096-025-05374-4)
Supplement: Supplementary file 1 — Supplementary file1 (DOCX 912 KB) [file 10096_2025_5374_MOESM1_ESM.docx]

**Supplementary Figures**


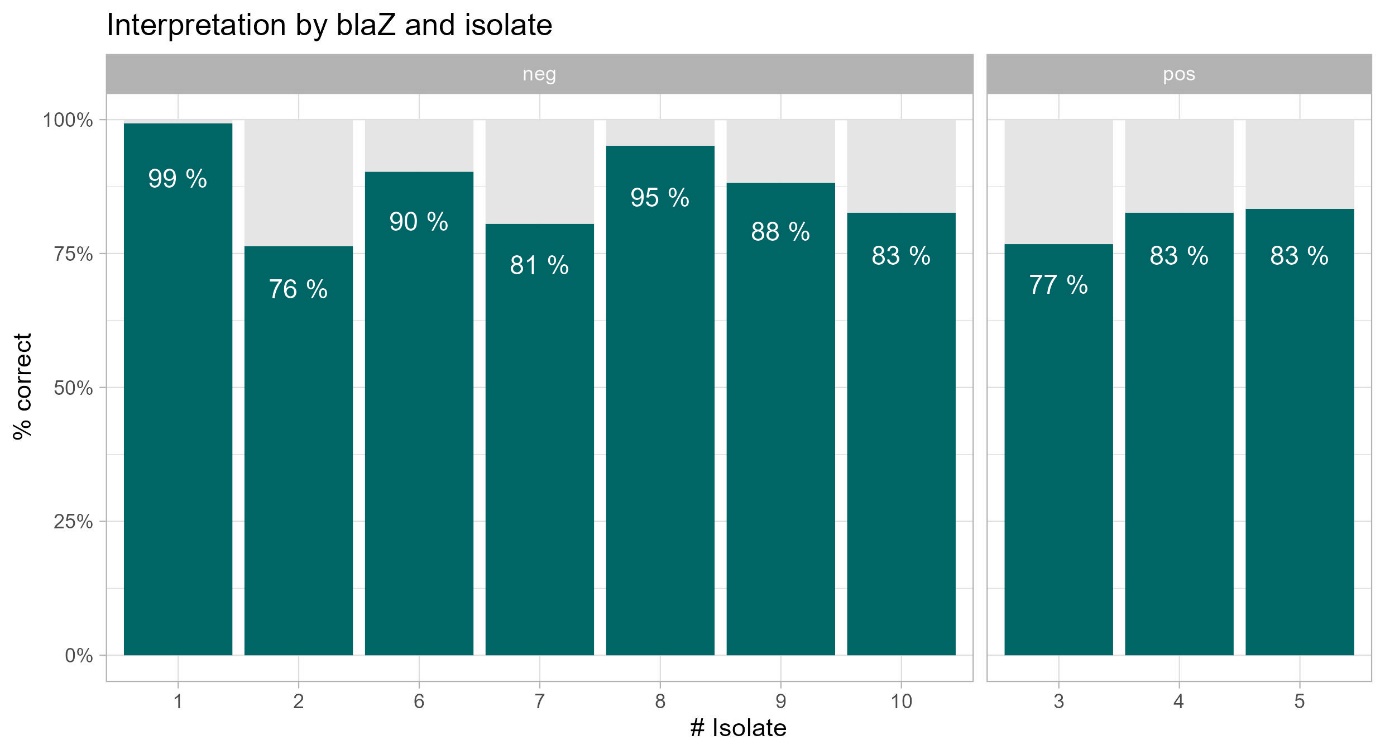


**Supplementary Figure 1:** Proportion of correct intrepretations (%) per isolate. Isolate 1 is the ATCC12600 strain.


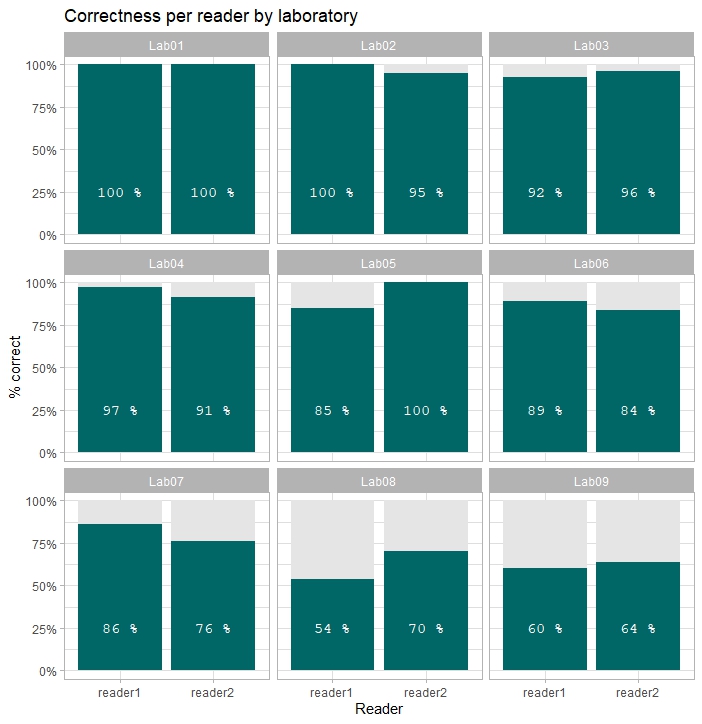


**Supplementary Figure 2:** Proportion of correct interpretations (%) between independent readers per laboratory.


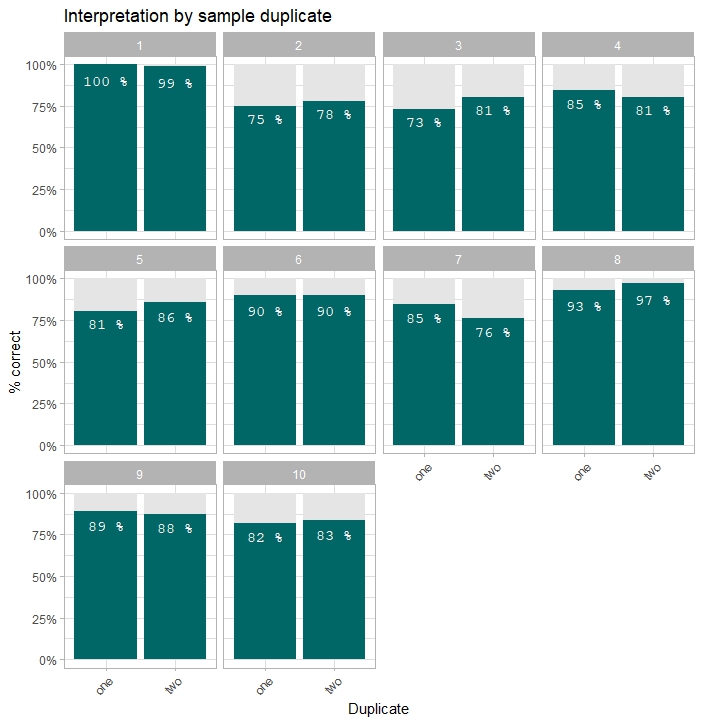


**Supplementary Figure 3:** Proportion of correct interpretations (%) per blinded sample duplicate. Each laboratory received each test strain as a blinded duplicate.


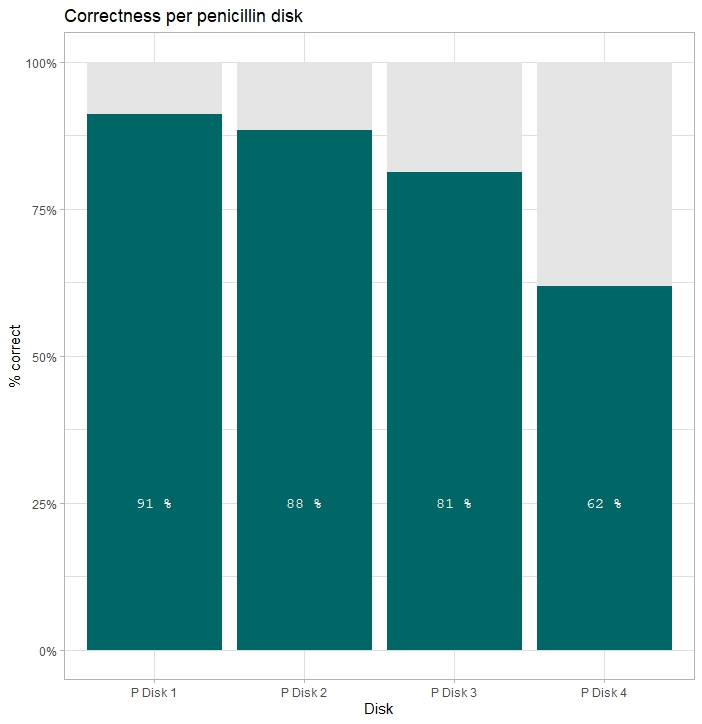


**Supplementary Figure 4:** Proportion of correct interpretations (%) depending on the benzylpenicillin disk used. Disk 1 = OXOID Penicillin G 1 Unit; Disk 2 = MAST Penicillin G 1 Unit; Disk 3 = SIRSCan®; Disk 4 = BD BBL™ Sensi-Disc™


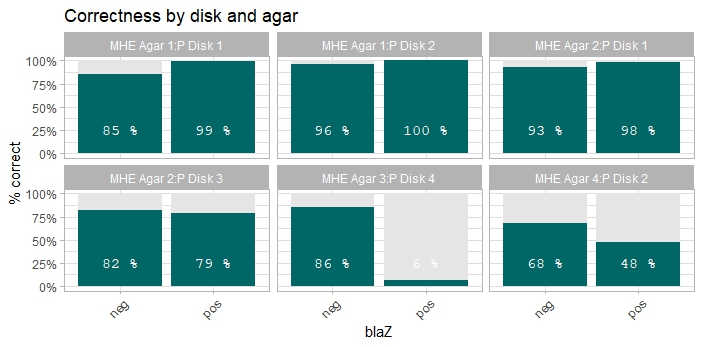


**Supplementary Figure 5:** Correctness according to disk and agar. The combinations of MH Agar 1 with P Disk 1 occurred in 2 laboratories, the combination MH Agar 1:P Disk 2 occurred in 3 laboratories. All other combinations were used in only one laboratory. MH agar 1 = BioMérieux Müller-Hinton-E-Agar; MH agar 2 = BD BBL Müller-Hinton II agar; MH agar 3 = BD BBL Müller-Hinton-II-Agar - in-house made; MH agar 4 = ThermoFisher Mueller Hinton agar. Disk 1 = OXOID Penicillin G 1 Unit; Disk 2 = MAST Penicillin G 1 Unit; Disk 3 = SIRSCan®; Disk 4 = BD BBL™ Sensi-Disc™


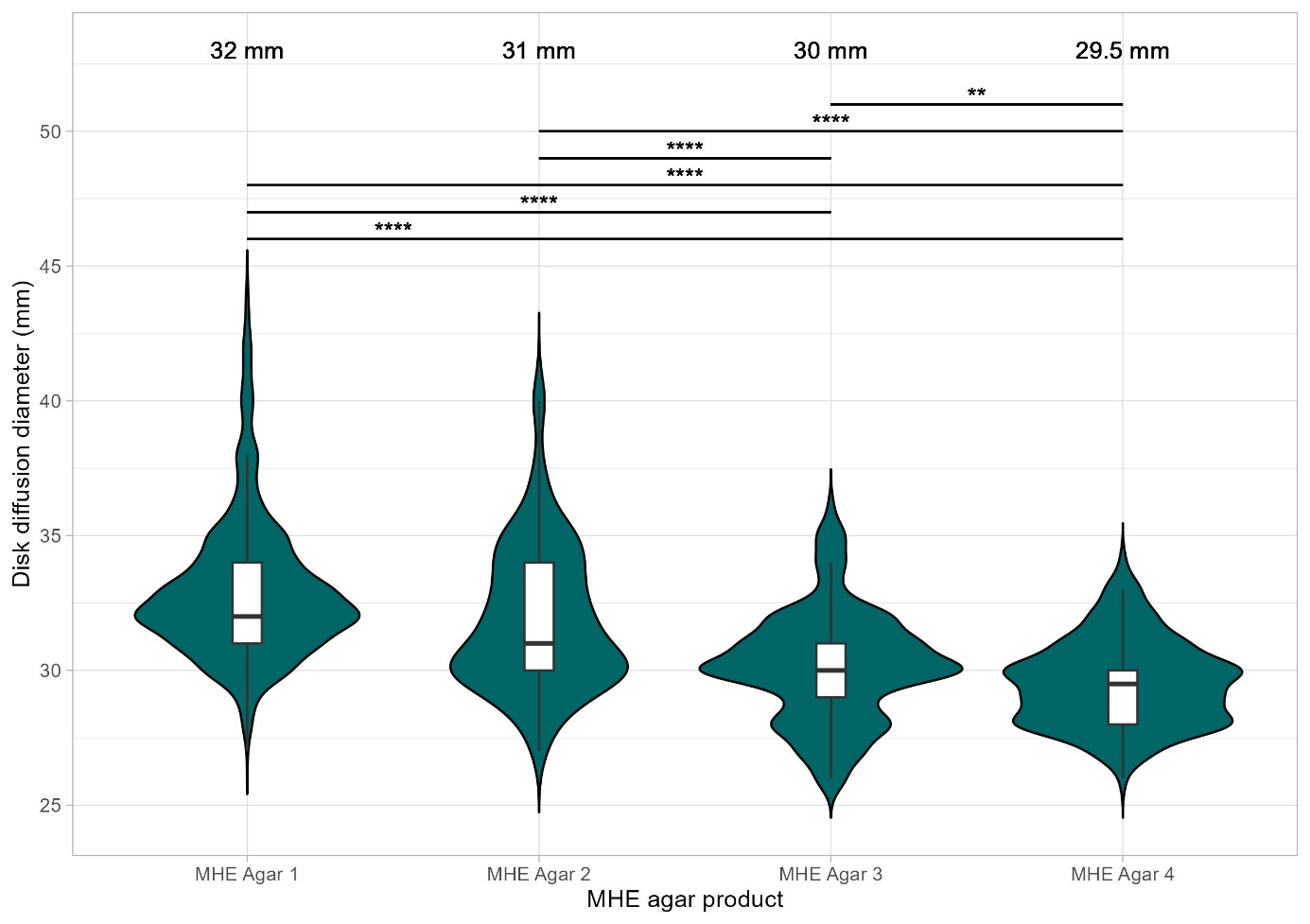


**Supplementary Figure 6:** Violin plot of disk diffusion zones per MHE agar. MH agar 1 = BioMérieux Müller-Hinton-E-Agar; MH agar 2 = BD BBL Müller-Hinton II agar; MH agar 3 = BD BBL Müller-Hinton-II-Agar - in-house made; MH agar 4 = ThermoFisher Mueller Hinton agar.


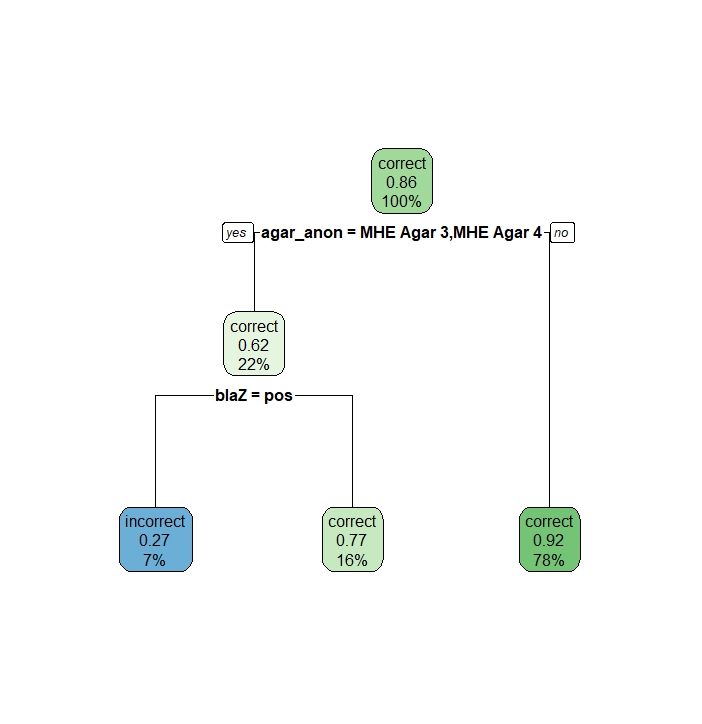


**Supplementary Figure 7:** Regression (decision) tree for variables separating correct from incorrect reads. The complexity parameter (cp) was set at 0.01. Using MH agar 3 or 4 vs. using MH agar 1 or 2 was the best variable in separating correct from incorrect reads.

**Simulation model of benzylpenicillin and penicillinase diffusion**

We modelled the spatial diffusion of benzylpenicillin from a point source (platelet) placed at position x_0_ = 0 and the subsequent inhibition by penicillinase produced by resistant bacteria. Diffusion of both benzylpenicillin $P\left( x \right)$and penicillinase $E\left( x \right)$was approximated by a simple exponential decay model:

$P\left( x \right)=e^{-\alpha r|x-x_{0}|}$ and $E\left( x \right)=e^{-\beta r|x-x_{e}|}$

$\alpha$ and $\beta$are the base decay rates for benzylpenicillin and penicillinase, respectively, modified by an agar diffusion parameter $r$. The position $x_{e}$ corresponds to the first location where benzylpenicillin concentration falls below the minimal inhibitory concentration (MIC).

The effective benzylpenicillin concentration after enzymatic inhibition was calculated as:

$$P_{eff}= \frac{P(x)}{1+r*E(x)}$$

Where $r$ is the inhibition strength parameter representing enzyme activity.

The MIC threshold was extended by a proportion to represent a “partial inhibition” range, in which some, but not all bacterial growth is prevented (i.e. corresponding to the “edge zone”).

In the simulation model, base decay rates $\alpha$ and $\beta$, agar diffusion factor $r$, inhibition strength $k$, MIC (and the size of the partial inhibitory range) can be set by user inputs. The program then visualizes the concentration curves over distance from the platelet of penicillin in the absence of penicillinase (blaZ negative isolate, blue curve) and effective benzylpenicillin in the presence of penicillase (blaz positive isolate, green curve).

The distance ranges from the platelet in which the respective benzylpenicillin concentration is within the partial inhibitory range is assumed to result in the “edge zone”. This edge zone is visualized as a color gradient from grey to black (mimicking the color gradient of the edge of the inhibition zone in a disk diffusion test). A short edge zone results in a sharper gradient, while a longer edge zone results in a “fuzzy” image.

Key assumptions of the model include: (i) diffusion is spatially one-dimensional and time-independent, (ii) both benzylpenicillin and penicillinase diffuse with constant exponential decay rates, (iii) the agar medium uniformly reduces diffusion rates via a multiplicative factor, (iv) penicillinase production is localised at the MIC threshold, and (v) inhibition follows a simple multiplicative reduction without saturation kinetics or feedback effects. The model does not incorporate detailed reaction–diffusion equations, enzyme turnover, or heterogeneous medium effects, but provides a simplified framework for exploring qualitative effects of parameter changes on inhibition zone size. Thus, the model should be understood as a simplified visualization of how different parameters can influence the fuzzy edge phenomena.


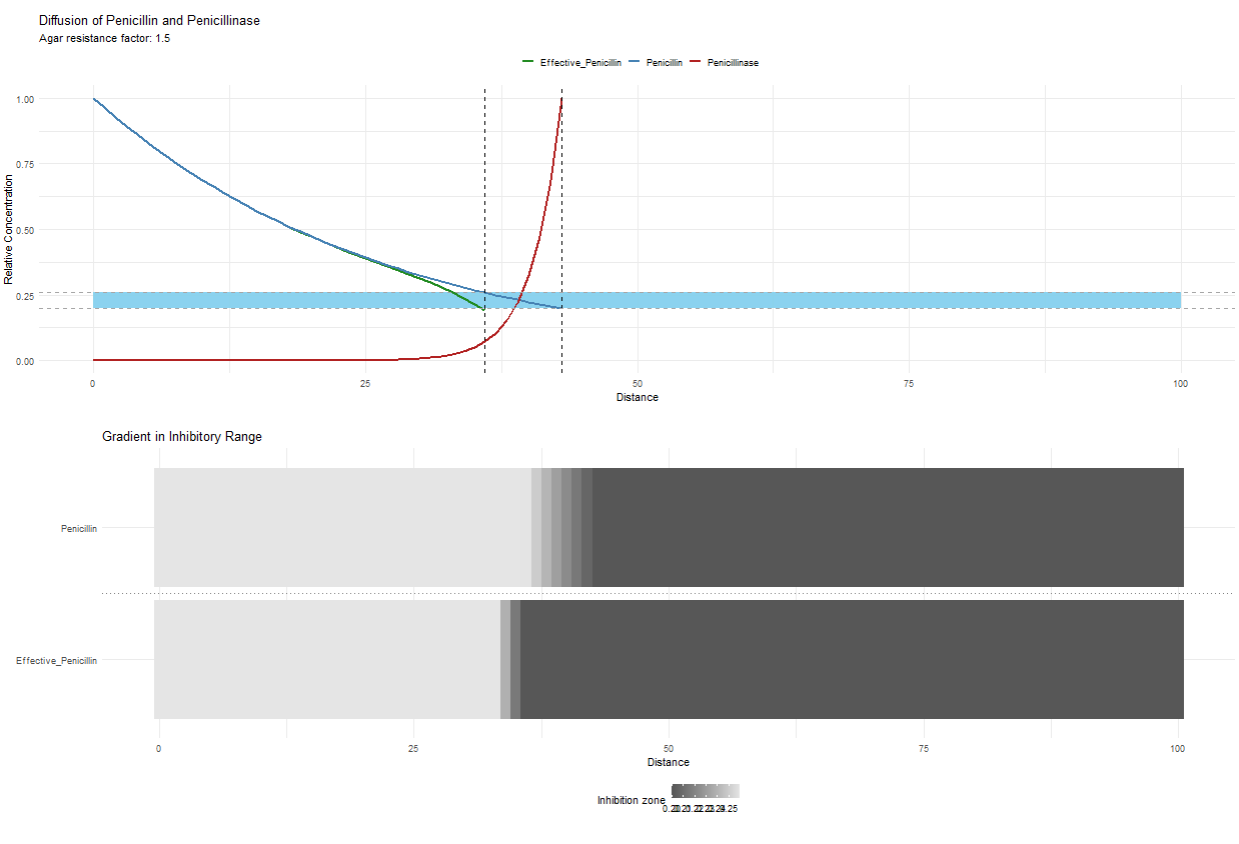


**Supplementary Figure 8:** Modelled zone edge on an agar with low agar resistance (i.e. high diffusion capacity). The blue line and upper bar (“penicillin”) represent an isolate without penicillinase activity. A broad transition zone (“fuzzy edge”) can be seen. The green line and lower bar (“effective penicillin”) represent an isolate with penicillinase activity. The transition zone is very narrow (“sharp edge”). The difference in the transition zone between both isolates is easy to distinguish.


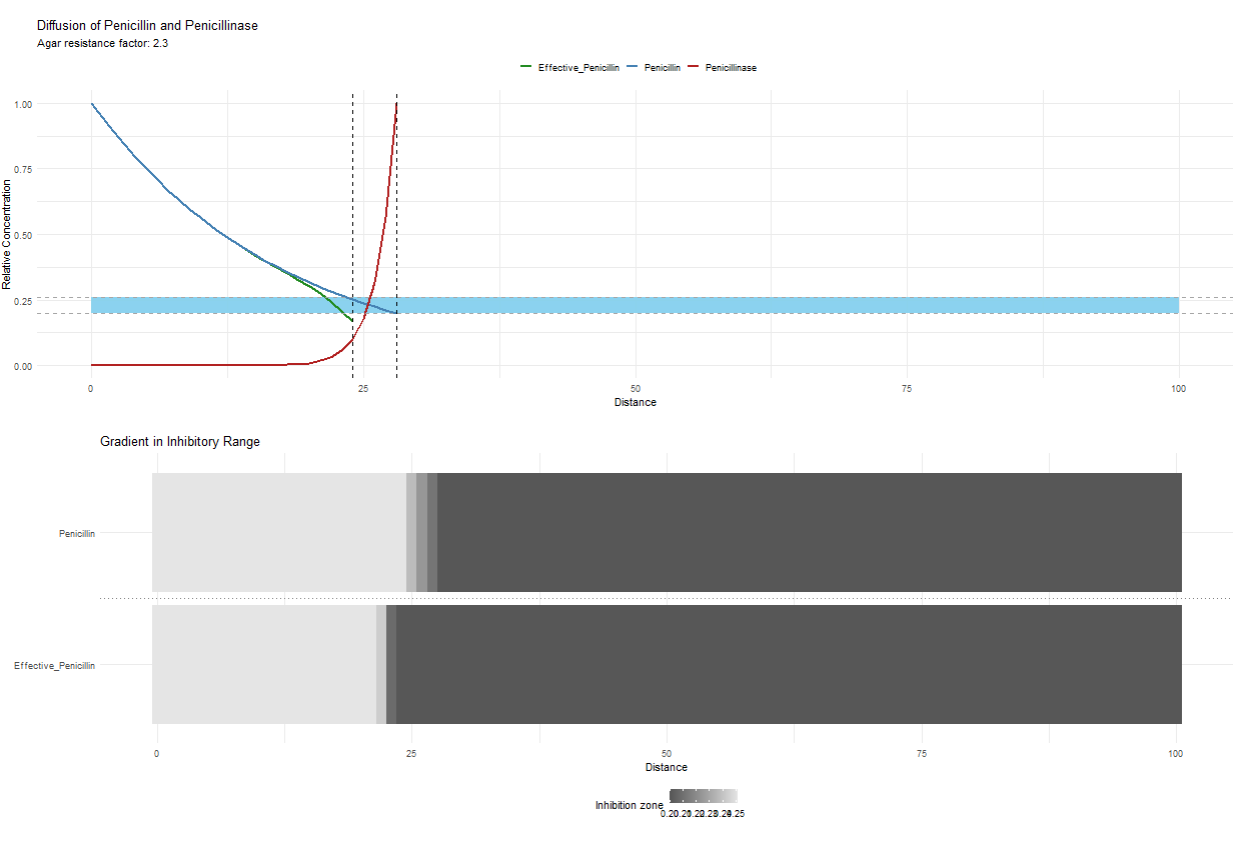


**Supplementary Figure 9:** Modelled zone edge on an agar with higher agar resistance (i.e. low diffusion capacity). The blue line and upper bar (“penicillin”) represent an isolate without penicillinase activity. A transition zone (“fuzzy edge”) can be seen. The green line and lower bar (“effective penicillin”) represent an isolate with penicillinase activity. The transition zone is very narrow (“sharp edge”). However, the difference in the transition zone between both isolates is difficult to distinguish compared to supplementary figure 5.
